# Corticosteroid therapy in regressive autism: a retrospective study of effects on the Frequency Modulated Auditory Evoked Response (FMAER), language, and behavior

**DOI:** 10.1186/1471-2377-14-70

**Published:** 2014-05-15

**Authors:** Frank H Duffy, Aditi Shankardass, Gloria B McAnulty, Yaman Z Eksioglu, David Coulter, Alexander Rotenberg, Heidelise Als

**Affiliations:** 1 Department of Neurology, Boston Children’s Hospital and Harvard Medical School, 300 Longwood Avenue, Boston, Massachusetts 02115, USA; 2 Department of Psychiatry (Psychology), Boston Children’s Hospital and Harvard Medical School, 320 Longwood Avenue, Boston, Massachusetts 02115, USA; 3 Pediatric Neurology, Golisano Children’s Hospital and Upstate Medical University, 90 Presidential Plaza, Syracuse, New York 13292, USA

**Keywords:** Autism, Behavior, Corticosteroids, Distortion, EEG, Electroencephalogram, Evoked potential, EP, Frequency modulated auditory evoked response, FMAER, Language, Regression, Spectral, Steroids, STG, Superior temporal gyrus, Treatment

## Abstract

**Background:**

Up to a third of children with Autism Spectrum Disorder (ASD) manifest regressive autism (R-ASD).They show normal early development followed by loss of language and social skills. Absent evidence-based therapies, anecdotal evidence suggests improvement following use of corticosteroids. This study examined the effects of corticosteroids for R-ASD children upon the 4 Hz frequency modulated evoked response (FMAER) arising from language cortex of the superior temporal gyrus (STG) and upon EEG background activity, language, and behavior. An untreated clinical convenience sample of ASD children served as control sample.

**Methods:**

Twenty steroid-treated R-ASD (STAR) and 24 not-treated ASD patients (NSA), aged 3 - 5 years, were retrospectively identified from a large database. All study participants had two sequential FMAER and EEG studies;Landau-Kleffner syndrome diagnosis was excluded. All subjects’ records contained clinical receptive and expressive language ratings based upon *a priori* developed metrics. The STAR group additionally was scored behaviorally regarding symptom severity as based on the Diagnostic and Statistical Manual IV (DSM-IV) ASD criteria list. EEGs were visually scored for abnormalities. FMAER responses were assessed quantitatively by spectral analysis. Treated and untreated group means and standard deviations for the FMAER, EEG, language, and behavior, were compared by paired t-test and Fisher’s exact tests.

**Results:**

The STAR group showed a significant increase in the 4 Hz FMAER spectral response and a significant reduction in response distortion compared to the NSA group. Star group subjects’ language ratings were significantly improved and more STAR than NSA group subjects showed significant language improvement. Most STAR group children showed significant behavioral improvement after treatment. STAR group language and behavior improvement was retained one year after treatment. Groups did not differ in terms of minor EEG abnormalities. Steroid treatment produced no lasting morbidity.

**Conclusions:**

Steroid treatment was associated with a significantly increased FMAER response magnitude, reduction of FMAER response distortion, and improvement in language and behavior scores. This was not observed in the non-treated group. These pilot findings warrant a prospective randomized validation trial of steroid treatment for R-ASD utilizing FMAER, EEG, and standardized ASD, language and behavior measures, and a longer follow-up period.

Please see related article http://www.biomedcentral.com/1741-7015/12/79

## Background

Autism Spectrum Disorders (ASD) or Autism refers to a complex range of developmental disorders that are characterized by impairments in language difficulties with social interactions, and often rigid, repetitive and/or stereotypical behaviors and interests. The incidence of autism has increased over the last few decades, with as many as one in 88 children identified with ASD [1].

Retrospective studies indicate at least three distinct patterns of symptom onset in autism. In the most common form symptoms of autism are evident early in infancy. In the second form, an initial period of typical development is followed by an unexpected cessation or significant slowing in the continued acquisition of communication and/or social skills and the child reaches a developmental plateau. In the third and perhaps most intriguing form, often referred to as regressive autism, a period of normal or near normal early development is followed by a cessation of all further development and loss of previously acquired communication and/or social skills, most often of both [2]. In this third form, developmental regression usually occurs between 15 to 30 months of age and can occur rapidly over a very few days, or more slowly in the course of several weeks or even months. Once development has regressed, such children typically follow the standard ASD developmental profile [3,4]. Ozonoff et al. point out that although “more children may present with a regressive course than previously thought parent report methods do not capture this phenomenon well” [5]. There may be confusion among early-starting slow regression, a developmental plateau, and frank, abrupt regression defined by rapid loss of previously achieved cognitive, language, communication, and behavioral milestones. Thus, anyone who studies patients with regressive autism must define carefully the parameters of regression applicable to the study.

ASD and in particular regressive autism have attracted worldwide attention. Prominent and abrupt loss of milestones typically results in referral to a child neurologist in order to search for potentially relevant etiological factors and hopefully, for initiation of remediation. Neurological disorders associated with autism symptomatology include Fragile X syndrome, tuberous sclerosis, Rett’s syndrome, mitochondrial disorders, and a multiplicity of genetic anomalies [6]. Regression associated with the Landau-Kleffner syndrome (LKS, an epileptic encephalopathy) may also present in a manner similar to R-ASD [7,8]. An overnight sleep electroencephalogram (EEG) may be required to diagnose and/or rule out LKS, given that the finding of significant focal or generalized sleep-activated discharges is taken as diagnostic for LKS [9]. To date the cause of abrupt autistic regression often eludes identification, despite the fact that onset of regression may appear to follow a distinct event, such as trauma, illness, or immunization. This report focuses exclusively upon regressive autism of abrupt onset showing initial, obvious, rapid regression over a time period of a few days to maximally a few weeks, followed by continuing regression at a slower pace over the ensuing months until a stable low functioning state is reached. This phenomenon will be referred to as ‘R-ASD’ in the current paper. It is probable that such abrupt regressions do not characterize the full third of autistic children, who are reported to “show regression” occurring across varying lengths of time [5].

The terminology used to describe regressive autism continues to be in flux. Given the similarity of presentation in children with R-ASD and children with early onset LKS the appellation ‘Landau-Kleffner Syndrome variant’ (LKSv) was used and often retained even when a definitive LKS diagnosis had been ruled out. In such cases many epileptologists object to the ‘LKSv’ terminology and increasingly utilize ‘regressive autism’ abbreviated as R-ASD, with focus upon the prominent autistic behavioral profile following regression. However, few R-ASD patients are evaluated early-on by ‘gold standard’ measures such as the ADOS and ADI [10,11]. Some behavioral neurologists object to the ‘autism’ appellation despite the evidence that most such children eventually receive that diagnosis [3,4]. For this paper, the abbreviation R-ASD is used.

Referring physicians often enquire about the utility of adrenal corticosteroids or glucocorticoids to treat patients with R-ASD. A literature review provides three possible justifications for this therapeutic approach. *First* are the various similarities of R-ASD and LKS; many neurologists consider corticosteroids the ultimate treatment for LKS when anticonvulsants have failed [12,13]. The R-ASD - LKS similarities considered include shared behavioral presentation [7,8], strong overlap of genetic, genomic and molecular networks [14], and in some older R-ASD patients magnetoencephalography (MEG) detected seizure discharges localized to the bilateral superior temporal gyri (STG) [15], which are the same brain areas that show ‘active’ EEG detected discharges in LKS [16]. Furthermore, in addition to LKS, several other forms of refractory epilepsy have been successfully treated using adrenal corticosteroids [17-19]. Given these similarities in underlying pathophysiology and genetics, and the potential of successful treatment, there is reason to hypothesize that the two syndromes may also share a positive response to corticosteroids. *Second*, it has been speculated that regressive autism might be an inflammatory or auto-immune disorder [20-22]. As corticosteroid administration constitutes an important treatment modality for the common auto-immune diseases [23], it is reasonable to consider steroids as potentially useful for R-ASD. A notable single case study demonstrated autistic language regression concomitant with an autoimmune disease; institution of steroid treatment of the autoimmune proliferative syndrome was associated with a parallel improvement in autistic symptoms particularly in language [24]. *Third,* several additional case studies reported in the literature also indicate positive effects of steroids in children with R-ASD [15,24,25].

The current study constitutes a preliminary investigation based on the retrospective review of steroid treatment of R-ASD pediatric patients at the first author’s institution. Two important restrictions were imposed upon the retrospective R-ASD patient selection. *First* R-ASD patients were included only if by prior sleep EEG true LKS definitively had been ruled out. Although open to further evaluation, it is commonly accepted clinically that an overnight EEG sleep study and/or a daytime EEG with a significant sleep epoch that fails to demonstrate significant sleep activation of epileptiform discharges constitutes adequate information to rule out LKS [9]. *Second,* R-ASD patients were included in the current study if they had two sequential FMAER studies separated by at least six months and their premedication FMAER study was read as absent or distorted. The first author and his associates [16] previously described the utility of the FMAER in the study of childhood language disorders (developmental dysphasia, autism, and especially LKS). The 4 Hz FMAER produces a scalp recorded 4 Hz sine wave steady-state evoked response that arises in normal subjects from the bilateral STG as determined by source analysis. It is absent or distorted (non-sinusoidal appearance) in subjects with language disorders involving the STG. A normal FMAER, thus, would suggest a clinically apparent language abnormality that must have arisen from a cortical language processing disorder which originated outside of or beyond that of the STG. Such a language disorder did not qualify the patient for the current study. Treatment outcome measures in the current study included bias-free, quantitative assessment of change in the FMAER and internally-developed clinical assessment scores of language and behavior.

## Methods

All analyses were performed at a university affiliated (Harvard Medical School) academic medical center Boston Children’s Hospital (BCH). The Developmental Neurophysiology Laboratory (DNL), under the direction of the first author, maintains an archived comprehensive database of several thousand previously studied clinical patients and research subjects. This database includes unprocessed (raw) EEG and Evoked Potential (EP) data in addition to associated referral and clinical information. Typically, patients had been referred for neurophysiological testing in order to rule out epilepsy and/or sensory processing abnormalities by EEG and/or EP study. For the current project all subjects meeting specified criteria as described below were selected from this data base.

### Subjects

#### Steroid Treated Autism with Regression (STAR)

The target group of study subjects selected from the DNL database was limited to children aged 3 – 5 years old diagnosed by academic child neurologists, psychiatrists and/or psychologists at BCH and other Harvard affiliated hospitals and allied medical facilities as having autism with a historically documented period of regression at onset. Regression was defined as the loss of age appropriate language, communication, cognitive ability, and behaviour determined by the referring physician and confirmed by the treating neurologist. Patients with a slowly developing plateau or a very slow regression over a prolonged period of time, as opposed to an abrupt regression, were not included in the STAR group. Furthermore, the target group was restricted to those patients, who were clinically treated with corticosteroids (STAR group) subsequent to an initial neurophysiological study that showed an absent or distorted FMAER, and who all had a second neurophysiological study after the treatment period was concluded, i.e. after at least six and not more than 36 months. All of the children received steroid therapy and/or other potential treatments exclusively on the basis of the clinical decision made by their treating neurologist in collaboration with the parent(s) responsible for the respective patient’s care. The treatment decisions were made much earlier than and completely independently of the current study. Post hoc record review revealed that none of the STAR group children received any additional pharmacological, behavioural, or educational interventions during the steroid treatment period.

#### Non-Steroid-Treated Autism (NSA)

The comparison group of subjects was similarly selected from the DNL database of 3–5 year old children with a diagnosis of autism with or without a documented regression similarly established as above by an academic clinician. They had not received steroid treatment at any time during the study period (NSA) nor had they received any other pharmacological treatment. Similarly to the STAR group children, they had two sequential neurophysiological studies, which were separated by at least six and not more than 36 months, each of which contained an EEG and an FMAER [16]; the first FMAER study showed an absent or distorted FMAER wave form. Clinical treatment of the NSA group children as for the STAR group, was in every case a decision between the respective treating physician and the parent(s), responsible for the child’s care.

### Subject exclusion criteria

For both the STAR and NSA study groups, exclusion criteria included: (1) Co-morbid neurologic syndromes that may present with autistic features (for example, Rett's, Angelman’s and Fragile X syndromes, tuberous sclerosis, or mitochondrial disorders); (2) clinical seizure disorders or EEG reports suggestive of an active seizure disorder or epileptic encephalopathy such as the Landau-Kleffner syndrome or prominent discharge activation during drowsiness or sleep (Note: occasional EEG spikes were not an exclusion criterion) [26]; (3) a primary diagnosis of global developmental delay (GDD) or developmental dysphasia; (4) other concurrent neurological disease processes that might induce EEG alteration (for example hydrocephalus, hemiparesis or known syndromes affecting brain development); (5) significant primary sensory disorders for example, blindness and/or deafness (6); use of anticonvulsants (e.g. valproate, levetiracetam) at the time of the first study (Note: Prior failed use of a medication such as valproate did not constitute an exclusion criterion [27]); (7) inadequate or incomplete clinical information; and (8) a normal initial FMAER test result. All subjects in the DNL ASD data base, who fulfilled the study’s in-and exclusionary criteria were included in the study sample. This yielded a study population of 20 target (STAR) and 24 comparison group (NSA) subjects.

### Steroid treatment management of STAR patients

Before starting medications the treating child neurologist presented and discussed the risks and potential benefits of steroid medications (http://www.mayoclinic.com/health/steroids/HQ01431) with the child’s parents. When agreed to by the parent in each case the primary pediatrician was contacted and agreement obtained for the management of the necessary testing to include at a minimum: (1) Weekly stool guaiac tests; (2) weekly (or more frequently as indicated) blood pressure measurements; (3) weekly urine glucose tests; (4) periodic blood sugar and electrolyte assessments; and (5) willingness to manage any attendant complications. Often Visiting Nurses performed these tests at the patients’ homes and most parents were successfully trained in obtaining reliable blood pressure measurements. All parents successfully obtained the necessary weekly weight measurements. Oral prednisolone (Prelone™ or Orapred™) was administered by the parents on a daily basis at 2 mg/kg/day. Dosage was occasionally down-adjusted on the basis of minor complications.

At the monthly neurology office visits laboratory findings were reviewed presence of complications assessed, and clinical signs of potential increased intracranial pressure evaluated (appearance of optic discs, quality of eye movements, deep tendon reflexes, Babinski responses, suppleness of neck, and level of consciousness). Ocular lenses were viewed for potential development of cataracts. Language changes from the previous visit were recorded, as measured by use of the Clinical Language Status Questionnaire (CLSQ), (see below). If there was no evidence of language improvement after four months of treatment (considered an ineffective treatment response) or if initial monthly incremental improvement stabilized for two consecutive months (considered a response plateau), a gradual medication taper was instituted.

### Institutional review board approvals

All subject data were retrospectively evaluated under a protocol approved by the Institutional Review Board (IRB) Office of Clinical Investigation, BCH. The protocol was in full compliance with the Helsinki declaration, and solely required de-identification of all personal information related to the already collected clinical data without requirement of informed consent.

### Data acquisition

#### ***Neurophysiology recording conditions data collection, and initial data processing***

All subjects’ electrophysiological data utilized in this study were gathered by a registered EEG technologist in the Clinical Neurophysiology Laboratory of BCH from 30 scalp channels via gold cup electrodes applied with collodion after careful measurement. A 31^st^ channel carried the FMAER trial marker. A 32^nd^ channel carried eye movement and blink artifact information. Data were digitized at 256 Hz after amplification by a Cardionics™ 32 channel EEG amplifier (Cardionics Inc. 910 Baystar Blvd Webster, TX 77598 USA) set to 1–100 Hz pass band. The 30 EEG scalp channels used included the following: FP1, FP2, F7, F3, FZ, F4, F8, FC5, FC1, FC2, FC6, T7, C3, CZ, C4, T8, CP5, CP1, CP2, CP6, P7, P3, PZ, P4, P8, O1, OZ, O2, TP9, and TP10 [28]. Data for the FMAER were gathered over 5–20 minutes with additional time allowed for rest breaks as indicated. The patient and a parent, when behaviorally indicated, were together within view of the technologist through a one-way mirror window in a sound-shielded room, adjacent to the recording equipment. Off-line, the EEG data and accompanying trial markers were visually evaluated and epochs containing excessive eye-blink, muscle and movement artifact were marked for removal from subsequent analysis. All 30 channel data were visually inspected for EEG abnormalities and for creating the common average reference. For the current study, FMAER analysis was restricted to 14 active scalp electrodes: F7, F8, T7, T8, P7, P8, TP9, TP10, F3, F4, C3, C4, P3, and P4.

#### ***Frequency Modulated Auditory Evoked Response (FMAER)***

Language comprehension requires decoding of rapidly changing speech streams; detection of FM within speech has been hypothesized as essential for accurate phoneme detection and word comprehension [16]. The FMAER was developed as a steady-state EP to assess the brain’s response to rapid changes in the frequency modulation (FM) of an applied auditory stimulus in extension of and based upon the pioneering work of Green Stefanatos and others [15,29-33]. Recently the FMAER has been documented as showing unilateral or bilateral abnormalities in the developmental dysphasias, Landau-Kleffner syndrome, and in some subjects with autism. Moreover, a previously absent FMAER has been reported to appear after successful treatment of LKS. By source analysis, the FMAER has been shown to arise bilaterally from the superior temporal gyri (STG) of neuro-typical subjects. The orientation of the STG source dipoles in normal subjects is such that they each point at one end of the dipole toward the midline frontal region and at the other end to the ipsilateral inferior, posterior temporal region. The best electrodes to record an ipsilateral signal when using the common average reference are TP9 (left posterior-inferior temporal for left source dipole) and TP10 (right posterior-inferior temporal for right source dipole). Midline frontal electrodes (FZ, FC1, FC2) usually manifest bilateral overlapping source dipole projection. The central electrodes (C3-left central, C4-right central) give the best view of the corresponding hemisphere’s source dipole’s superior-anterior projection. The FMAER may be normal, or as in pathology, of low amplitude, distorted, or absent. In rare cases of pathology the FMAER source location may be outside the usual STG location and/or the source dipole may point in unexpected directions [16].

As previously detailed [16] the FMAER was formed from a carrier sine wave at 1000 Hz frequency modulated by a slower 10 Hz sine wave causing the frequency of the carrier wave to shift (“deviate”) between 960 and 1060 Hz at the 10 Hz rate, producing a warbling tone. The 10 Hz sine wave was then amplitude modulated by a slower 4 Hz sine wave such that the warbling (FM modulation) was sinusoidally turned on and off (AM modulated) at the 4 Hz rate. This process caused the 10 Hz “warbling” of the 1000 Hz sine wave carrier to be sinusoidally turned fully on and off (100% modulation) at 4 Hz. By setting a trigger pulse to the start of each second of 4 Hz signal, signal averaging was performed in order to obtain the 4 Hz steady-state FMAER - time locked to the 4 Hz AM modulation of the 10 Hz FM modulation, i.e., to the turning on and off of the FM. Between 500–1000 trigger pulses were averaged over an epoch of 1000 msec using BESA software (BESA GmbH, Freihamer Str. 18, 82116 Gräfelfing, Germany). The one-second-long steady state FMAER tracing from neurotypical subjects manifests a 4 Hz sine wave. The stimulus’ sound pressure level was held at approximately 78db Sound Pressure Level (SPL), measured at the ears and was delivered either by earphones or by nearby bilateral speakers depending upon the environment and subject preference and tolerance. At time of clinical study, FMAERs were initially formed from successive thirds of all stimuli which, when separately evaluated, allowed assessment of response consistency. If responses were similar across all three thirds, a global average wasformed for interpretation. If such consistency was not observed, more data were collected to improve the signal to noise ratio. The Chirp2™ Signal Generator (Mind Spark Inc., 172 Washington St, Newton, MA 02458 USA), a small stand-alone battery operated device, was employed to perform all aspects of the FMAER from signal generation through trial marker formation.

FMAER data were viewed on BESA software using the common average reference since mastoid/ears references induce artifactual spatial localization. Each subject’s response was visually reviewed for both hemispheres. The normal response consisted of a clear 4 Hz sine wave. An abnormal response varied from absence of any obvious response to a distinctly non-sinusoidal response suggesting a mix of multiple frequencies. In order to quantify responses for analytic purposes power spectral analysis was performed on the FMAER traces (BESA software) and the resulting 4 Hz spectral value was utilized as the primary quantitative measure of the brain’s response [16].

### Noise analysis

The steady state 4 Hz FMAER when ‘absent’ by visual inspection rarely presents as a simple flat-line display. Typically a low amplitude epoch of apparently random noise is visualized that does not appear to contain an obvious 4 Hz component. Such a noisy response reflects four possibilities: (1) There is no 4 Hz response and the noise reflects incomplete signal averaging; (2) There is a low amplitude 4 Hz response which is masked by noise from incomplete signal averaging; (3) The response is distorted showing frequencies adjacent to 4 Hz (side-band distortion) which causes the response to appear non-sinusoidal or noisy; or (4) A combination of the above three possibilities might be identified. In order to assess these possibilities in keeping with established techniques, a plus-minus averaging technique was utilized “in which measurements from every other trial are inverted prior to creating the averaged result which removes the consistent signal component by the alternating addition and subtraction (and) the noise component is the same as that produced by the standard average [34] (page 61)”. By creating the root-mean-square voltage (Vrms) of a plus-minus average one can compare this Vrms to the Vrms of the standard average. If there is no evoked response the standard and plus-minus averages typically show nearly the same Vrms values. If there is an evoked response of any sort the standard average typically produces a Vrms greater than does the plus-minus average. Since the plus-minus and the standard averages have the same noise distribution, spectral analysis of the plus-minus average can be subtracted from that of the standard average in order to estimate the spectral distribution of signal added in response to the stimulus. An advantage to the plus-minus technique is that noise is estimated on the very same EEG segments that are averaged in order to produce the FMAER. Software was developed in-house to perform plus-minus averaging and to create Vrms data.

For the current study noise analysis was limited to the STAR population’s FMAER data from the two left hemisphere electrodes (TP9 C3), which manifested the most significant pre- and post-treatment FMAER difference at 4 Hz (Table 1). The locations roughly correspond to the maximum scalp projection of the superior and inferior aspects of the typical FMAER dipole generator located in the left superior temporal gyrus [16]. At the time of signal averaging, the EEG was additionally band pass filtered from 1–12 Hz. An estimate of the FMAER response was taken at 4 Hz and an estimate of ‘side-band noise’ was taken as the average of the spectral data at 2, 3, 5, 6, and 7 Hz at both study points. To start, the Vrms of the standard average and plus-minus average for the first time point (before steroids) data were compared to determine if any added component was evident in the standard average. Next, and separately for the first and second study time point, spectral analysis was performed independently on the standard FMAER average as well as on the plus-minus average. The plus-minus average spectral results were then subtracted from the standard spectral results at each study point separately. The resulting spectral difference result estimated the portion of spectral signal attributable to the stimulus, after removal of the best estimate of spectral background noise.

**Table 1:** Noise analysis of the FMAER for Time 1 and Time 2, STAR group


(a) Time one pre-treatment data: Standard vs. Plus-Minus FMAER									
Electrode	Vrms	4 Hz		5 Hz		6 Hz		2, 3, 7 Hz	
	T	p	T	p	T	p	T	p	p
C3	+3.84	0.0012	+3.77	0.0014	+2.97	0.0083	+2.39	0.0278	n.s
TP9	+3.24	0.0046	+3.43	0.0030	+2.26	0.0388	n.s.		n.s
(b) Time post-treatment data: Standard vs. Plus-Minus FMAER									
Electrode	4 Hz	2, 3, 5, 6, 7 Hz							
	T	p	p						
C3	+5.96	0.0000	n.s						
TP9	+5.94	0.0000	n.s						
(c) Time 1 vs. Time 2: noise corrected spectral data									
Electrode	4 Hz								
	T	p							
C3	+3.03	0.0072							
TP	+3.69	0.0017							

### Subjective evaluation of EEG abnormality

All first and second EEG studies were visually reassessed by a pediatric electroencephalographer blinded to subject history and group identity. All EEGs were reviewed in randomly selected order with respect to group and study order. Each EEG was scored as showing in ascending order of abnormality (if present): paroxysmal sharp theta sharp waves, and spike or spike wave discharges. After this ‘blinded’ estimation was completed, EEG results were sorted into first and second EEG study order, without group identity; subsequently the electroencephalographer compared each subject’s two sequential EEGs and classified their scores as showing ‘worsening’ ‘no change’, or ‘improvement’ between the first and second study. For example, a subject showing ‘spikes’ on the first study and ‘no spikes’ on the second study would be considered as ‘improved’.

### Language assessment STAR Group

STAR group subjects were followed by their child neurologists using a clinical language assessment referred to as the ‘Clinical Language Status Questionnaire (CLSQ)’ which was developed in-house well prior to the current study. It is as yet unpublished. The assessment was designed to assist pediatric neurologists involved in the pharmacological treatment of neurological disorders, in estimating language progress or lack thereof - from a child’s parent(s)’ report and the clinician’s direct assessment at the time of a clinical visit. It involves evaluation of both the child’s current best expressive and receptive language performance. In the current study, documentation of lack of response improvement or plateau, along with assessment of medical complication(s), constituted the primary evidence used for treatment discontinuation. Table 2 shows the CLSQ score definitions for expressive and receptive language performance. The term ‘Appears normal’ (Table 2) was defined as the clinician’s and family’s report of normal, age-appropriate speech. None of the children in this study received this score. ‘Appears nearly normal’ was defined as context and age appropriate speech with evidence of mispronunciations, poor or odd word choice, unusual fluency or unusually sparseness, unusual grammatical errors such as errors in tense , pronoun gender match and/or pluralization, and/or requirement of adult speech simplification in order to assure comprehension relative to age expectancy. The difference between ‘short meaningful 1–2 word phrases’ and “meaningful 3+ word phrases’ is self-explanatory. Although many well developed language tests exist, as documented by the American Speech-Language-Hearing Association (http://www.asha.org/assessments.aspx), most tend to be detailed yet too coarsely grained to capture this population’s limited language range. They are also typically too time-consuming for repeated administration by neurologists during recurring, clinical check-in visits. In contrast, the CLSQ may be completed in less than ten minutes. An additional advantage of the CLSQ for the current study was its comprehensiveness in capturing the full - while limited - range of language change observed in this patient population. Parents are shown short phrases (without the numerical scores) and asked to identify their child’s current relevant expressive and receptive status. The neurologist stands by to clarify, assist, and help resolve two caregivers’ divergent opinions. Neurologists using the CLSQ are sensitive to the language performance definitions in question. The study subjects’ CLSQ score assignments occurred without prior knowledge of the current study.

**Table 2:** **Clinical Language Status Questionnaire (CLSQ)**

**Expressive score**	**Receptive score**
10 Appears normal	10 Seems normal
9 Normal but dysarthric	9 Nearly normal receptively
9 Nearly normal expressively	9 Responds to incidental language
9 1–3 word sentence	9 Responds to multiple (>2) part requests
9 Produces meaningful (>2 word) phrases	8 Responds to two part requests
8 Produces meaningful 1–2 word phrases	6 Responds to one part requests
7 Produces single words on own initiative	4 Responds to words without gestures
5 Mimics words strings without meaning	2 Responds to words with gestures
4 Produces meaningless words	1 Responds better to voices than to noises
3 Only sings words	0 Responds better to noises than to voices
1 Produces word-like meaningless sounds	0 Acts deaf
1 Babbles, no words	
0 Makes noises, or only screams	
0 Mute	

Receptive Language Scoring

Ask the parent(s): “In the course of the last month what is the most complex spoken language, given without helpful gestures, that you know your child understands and may respond to but need not respond to every time.”

Ask for discrete examples and match to 11 shown possibilities. We are after the highest level response of which the parents are certain. Parents may be shown the alternative choices but not the associated scores.

Expressive Language Scoring

Ask the parent(s): “In the course of the last month what is the most complex spoken language you have heard your child produce.” Again we are after the highest level of language production of which the parents are certain. Match to one of the 14 shown possibilities. Parents may be given the alternative choices but not the associated scores.

### Language assessment NSA Group

Since the CLSQ was not employed for those pediatric populations who did not receive pharmacological intervention(s) the NSA group subjects lacked the CLSQ scores. Their language status was estimated retrospectively on the basis of the clinical assessment of expressive and receptive language abilities performed within the standard comprehensive neurological evaluation included in every office visit. The clinical reports closest in time to the two neurophysiological studies were utilized to score separately receptive and expressive language performance and to assign a change score from the initial to the second visit. The change score was scaled from minus (−) 2 to plus (+) 2 as follows: −2 = marked worsening; −1 = some worsening; 0 = no change; +1 = some improvement; +2 = marked improvement. In the case a clinician failed to distinguish between receptive and expressive language performance the same score was assigned to both categories. Failure to comment on language performance at all resulted in exclusion of the subject from consideration for the current study. No subjects were excluded on this basis. NSA subjects’ language scoring was performed well before the current study was undertaken and without knowledge as to which particular subject might be included in the study. Scorers had no knowledge of the current study’s goals or design the future group status of subjects, or the subjects’ FMAER results. Approximately three times as many children’s language reports were scored retrospectively as were declared eligible for inclusion in the study.

### Behavioral assessment

The DSM-IV criteria for Autistic Disorder (299.0) [35] aside from employment for diagnosis, were scaled in terms of severity for each STAR group child and the sum of the scaled scores was assigned as behavioral score. The direction of scaling was defined such that the higher the score was, the more severe the behavioral manifestations of ASD were. The symptom items and scaling are described in Table 3. Note, that item A2b) was omitted from the total score since none of the study children demonstrated “adequate speech”. Summed scaled item scores were assigned in order to quantify behavioral change in analogy to language change. For the current study scores were selected for the office visit nearest to the first and the second EEG/FMAER studies respectively. Scorers had no knowledge of the current study’s goals or design, the future group status of subjects, or the subjects’ FMAER results. The NSA group subjects lacked the DSM-IV based behavior scores.

**Table 3:** DSM-IV Criteria for Autism Disorder, and Scoring

**Criteria:**	**A subject must have a total of six (or more) items from A1, A2, and A3. with at least two from A1 and at least one each from A2 and A3**
A1.	Qualitative impairment in social interaction, as manifested by at least two of the following:
A1a)	Marked impairment in the use of multiple nonverbal behaviors such as eye-to-eye gaze, facial expression, body postures, and gestures to regulate social interaction.
A1b)	Failure to develop peer relationships appropriate to developmental level
A1c)	Lack of spontaneous seeking to share enjoyment, interests, or achievements with other people (e.g., by lack of showing, bringing, or pointing out objects of interest)
A1d)	Lack of social or emotional reciprocity
A2.	Qualitative impairments in communication as manifested by at least one of the following:
A2a)	Delay in, or total lack of, the development of spoken language (not accompanied by an attempt to compensate through alternative modes of communication such as gesture or mine)
A2b)	In individuals with adequate speech, marked impairment in the ability to initiate or sustain a conversation with others (omitted from scoring – see text)
A2c)	Stereotyped and repetitive use of language or idiosyncratic language
A2d)	Lack of varied, spontaneous make-believe play or social imitative play appropriate to developmental level
A3.	Restricted repetitive and stereotyped patterns of behavior, interests and activities, as manifested by at least one of the following:
A3a)	Encompassing preoccupation with one or more stereotyped patterns of behavior and restricted patterns of interest that is abnormal either in intensity or focus
A3b)	Apparently inflexible adherence to specific, nonfunctional routines or interests
A3c)	Stereotyped and repetitive motor mannerisms (e.g., hand or finger flapping or twisting, or complex whole-body movements)
A3d)	Persistent preoccupation with parts of objects
B.	A subject must show delays or abnormal functioning in at least one of the following areas, with onset prior to 3 years:
B1)	Social interaction
B 2)	Language as used in social communication
B3)	Symbolic or imaginative play
**Graded scoring – each item:**	(Item A2b omitted)
0	= absent
1	= possibly or very mildly present
2	= definitely present
3	= a very dominant characteristic
Overall score	= average of 14 scored items

### Data analysis

The BMDP2007™ statistical package (Statistical Solutions Stonehill Corporate Center, Suite 104, 999 Broadway, Saugus, MA 01906 USA) [36] was utilized for standard statistical analyses. Program 2D (P2D) was used for data description, Program 3D (P3D) for paired t-tests, and Program 2R (P2R) for multiple regression. Fisher’s exact test for 2 × 2 tables utilized an online Graph Pad™ program. Fisher’s exact test for 2 × 3 tables with Freeman-Halton extension utilized the online Statistical Calculators™ program.

## Results

### Group demographics

The STAR group’s sample size was 20 and the NSA group’s 24 subjects (see Table 4 for demographics). The means and standard deviations (SD) of the ages for the two groups at the time of the first neurophysiologic study were 3.91 (1.25) and 4.52 (1.80) years respectively and the mean and SD time between the first and second studies was 2.14 (1.61) and 1.90 (0.99) years respectively. Neither age at first study nor interval between studies differed statistically between the two groups. All STAR group subjects manifested language and behavior regression sufficiently abrupt to allow parents to date onset to within a few days to maximally a few weeks. Mean and SD STAR group age at regression onset was 18.93 (9.93) months and mean length of regression to a point of relative stability was 20.05 (12.7) weeks. Mean and SD of steroid treatment length was 9.125 (3.26) months with a range from 4 to 14 months. The two groups did not statistically differ by Fisher Exact test regarding gender or handedness (Table 4). By definition the STAR group was entirely (20 of 20) comprised of children with regressive autism. The NSA group contained, by chance, several children with regressive autism (7 of 24) (Table 4). This difference was significant by Fisher’s Exact test, p = 0.0001.

**Table 4:** Group demographics

	**STAR group (n = 20)**	**NSA group (n = 24)**	**t-test**	**p**
Age at first study (years)	3.909 +/− 1.248	4.522 +/− 1.800	1.29	n.s.
Time between studies (years)	2.136 +/− 1.609	1.904 +/− 0.989	0.49	n.s.
Age at regression (months)	18.925 +/− 9.928			
Length of regression (weeks)	20.053 +/− 12.70			
Length of treatment (months)	9.125 +/− 3.26			
Gender	18 males, 2 females	18 males, 6 females		n.s.
Handedness	18 right, 2 left	23 right,1 left		n.s.
Subjects with history of regression (n)	20 of 20	7 of 24		0.0001

### Change in FMAER between time 1 and time 2 subsequent to steroid treatment

As shown in Table 5 for the STAR group nine of 14 electrode measurements of the FMAER’s 4 Hz response manifested a significant change, measured by matched-paired t-test, between study values before and after steroid therapy. The biggest change was identified at the left posterior-inferior temporal electrode TP9 (p = 0.0037). In contrast, not one of the 14 analogous FMAER spectral measures compared between time 1 and time 2 for the NSA group was statistically significant. All NSA group members demonstrated an increase in 4 Hz magnitude from study 1 to study 2; this did not exceed the expected change with age. None of the NSA group’s study 1 to study 2 differences was significant at any of the electrode sites measured.

**Table 5:** Time 1 to Time 2 difference of the 4 Hz FMAER, paired t-tests

	**STAR group**			**NSA group**		
Electrode	Mean diff.	T	p	Mean diff.	T	p
F3	47.18	2.57	0.0188	16.83	1.17	n.s.
C3	24.15	3.02	0.0070	15.45	1.35	n.s.
P3	10.49	1.25	n.s.	3.23	1.04	n.s.
F7	3.38	0.51	n.s.	3.26	0.72	n.s.
T7	15.49	2.83	0.0106	6.31	0.83	n.s.
P7	65.08	2.37	0.0283	24.69	1.71	n.s.
TP9	56.91	3.31	0.0037	31.77	1.78	n.s.
F4	64.31	2.41	0.0260	20.89	1.21	n.s.
C4	22.66	3.12	0.0056	13.48	1.15	n.s.
P4	10.52	1.96	n.s.	3.12	0.59	n.s.
F8	18.47	1.44	n.s.	8.02	0.95	n.s.
T8	30.60	1.87	n.s.	1.08	0.07	n.s.
P8	77.83	2.86	0.0100	35.87	1.36	n.s.
TP10	83.49	2.41	0.0260	36.03	1.51	n.s.

T = t-test score; p = probability; n.s. = not significant;

Mean Diff. = mean of pre to post difference of 4 Hz FMAER spectral power, μV^2^/Hz.

**Figure 1:**
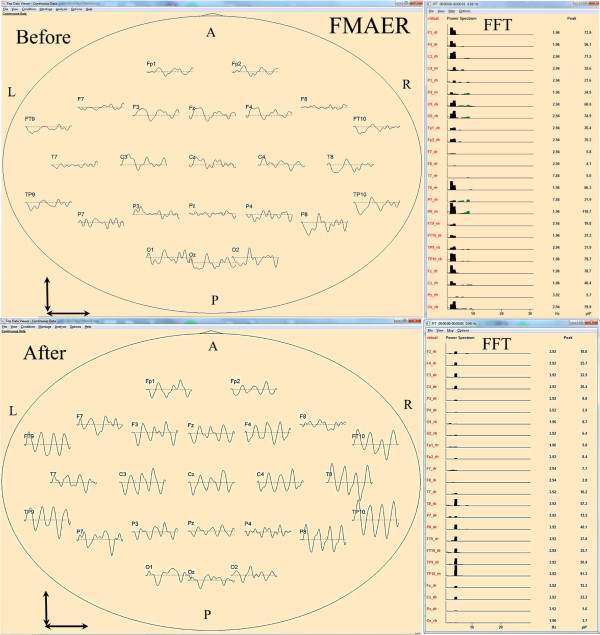
**FMAER and corresponding FFT, before and after steroids in regressive autism.** 4 Hz FMAER waveform data are shown within schematic ovals in vertex view with nose up, and left side of scalp to image left. The corresponding power spectra are shown to the immediate right. The top waveform and FFT displays were obtained prior to steroid administration. The bottom, corresponding displays were obtained after steroid administration. The vertical arrow to the lower left of each image represents 10 μV and the horizontal arrow beneath represents one second waveform length. The labels adjacent to the FMAER waveforms correspond to the standard EEG electrode 10–10 naming convention. Twenty-four electrodes’ waveforms are shown. The FFT power spectral data horizontal axis covers the 0–30 Hz range. Note the near absent 4 Hz FMAER waveform response before and excellent 4 Hz waveform response after steroid administration. Note the spread of spectral power over many frequencies in the FFT display before (above) which represents a distorted response. This contrasts to the nearly perfect 4 Hz response after steroid treatment (below) which shows little spectral spread (little distortion). For the vertex view display, waveforms are shown overlying their standard ‘10-10’ locations. For the FFT graphs, channel order from top to bottom is: F3, F4, C3, C4, P3, P4, O1, O2, Fp1, Fp2, F7, F8, T7, T8, P7, P8, FT9, FT10, TP9, TP10, Fz, Cz, Pz, Oz. The common average reference is utilized for the displayed data (a reference free or ‘rfr’ technique) [10]. Abbreviations: A = anterior, P = posterior, L-left, R = right, FMAER = 4 Hz frequency modulated auditory evoked response, FFT = fast Fourier transform - power spectrum analysis shown as μV^2^/Hz, μV = microvolt, Hz = Hertz or cycles per second.

For electrode locations see Figure 1.

Figure 1 illustrates an example of one STAR group subject’s relevant FMAER and associated spectral changes from before to after steroid administration: Note the absence of a clear 4 Hz sine wave following response before treatment in contrast to the excellent 4 Hz sine wave following response after treatment. The ‘before treatment’ fast Fourier transform (FFT) power spectrum response furthermore illustrates that the input 4 Hz stimulus appears at the cortex and demonstrates power at many different frequencies aside from the expected 4 Hz input response. This occurrence of spurious response frequencies in output to a single frequency input represents response distortion [37]. It is visualized in the FMAER waveforms by their non-sinusoidal appearance and in the FFT by the spread across the spectrum away from the primary 4 Hz driving frequency. Note however, that after steroids the FMAER waveform was sinusoidal at 4 Hz and the FFT showed a well-aligned response at the expected 4 Hz frequency without spectral spread, i.e. without distortion. All STAR subjects uniformly demonstrated such reduction of distortion after steroid treatment, while this change was not observed in the NSA group.

### Noise analysis

As summarized in Table 1 for the first study point, as shown in Table 1-(a), there was significantly increased Vrms in the standard average as compared to the plus-minus average at both electrodes selected (TP9 p ≤ 0.0046, C3 p ≤ 0.0012). Also at the first study point, both TP9 and C3 demonstrated significantly increased 4 Hz activity for the standard average as compared to the plus-minus average (TP9 p ≤ 0.0030, C3 p ≤ 0.0014). However, there was also spuriously increased 5 Hz activity (TP9 p ≤ 0.0388, C3 p ≤ 0.0083) and 6 Hz (C3 p ≤ 0.0278). These findings indicate that at the first study point in addition to an unexpected 4 Hz response there was also evidence of a strong noise response at frequencies other than 4 Hz, namely at 5 Hz and 6 Hz. In contrast, at the second study point, as shown in Table 1-(b), there was an even stronger 4 Hz response (TP9 p ≤ 0.0017, C3 p ≤ 0.0072), and moreover, this response now showed no evidence of significant accompanying noise responses at other frequencies. The 4 Hz FMAER response amplitude increase from time 1 to time 2, as shown in Table 1-(c) remained statistically significant when corrected for noise at approximately the same statistical significance level as shown in Table 5 for the uncorrected response.

### Change in EEG abnormality between STAR and NSA Groups

As demonstrated in Table 6 there was no significant difference across time points in the type of EEG abnormalities identified by visual inspection between the STAR and NSA groups by Fisher’s exact test. Thus, the children’s EEG, in contrast to the FMAER, failed to show any effect associated with of time or treatment.

**Table 6:** Difference between groups in EEG change from Time 1 to Time 2 EEG change summary

**Group**	**Worse**	**No change**	**Improved**	**by Fisher 2x3 exact test**
STAR	7	10	3	n.s.
NSA	4	12	8	

### Change in language function

As demonstrated in Table 7 the STAR group showed a significant difference in the mean CLSQ scores from time 1 to time 2; receptive and expressive language functions both showed a highly significant score increase i.e. improvement. For the NSA group the mean of the language change score for receptive language was 0.167 and for expressive language 0.542; neither reached statistical significance.

**Table 7:** Time 1 to Time 2 CLSQ difference scores for STAR group

**Language**	**Mean diff.**	**t**	**p**
Receptive	4.80	7.32	0.00001
Expressive	4.10	6.17	0.00001

t = t-test score; p = probability; n.s. = not significant; Mean Diff. = mean of pre- to post difference of CLSQ difference scores.

### Number of subjects showing change in language function

Negative language change scores indicated language worsening a score of zero indicated lack of change, and positive scores indicated language improvement from the first to the second visit. Table 8 shows two 3 × 2 Fisher’s exact tests for the number of subjects showing change (improvement, no change, or worsening) separately for receptive and expressive language. The results revealed improvement for a significantly higher number of STAR group subjects as compared to NSA group subjects, both in terms of receptive as well as expressive language.

**Table 8:** Time 1 to Time 2 Change in language scores between first and second study for STAR and NSA groups

	**Group**	**Better**	**NoDiff**	**Worse**	**Fisher exact**
Receptive language					
	STAR	17	3	0	P ≤ 0.0002
	NSA	6	16	2	
Expressive language					
	STAR	17	2	1	P ≤ 0.0031
	NSA	10	13	1	

p = probability; NoDiff = no difference.

### Language change for NSA subjects with and without history of regression

Table 9 compares receptive and expressive language change scores across the two time points for the number of NSA group subjects who had a history of regression with those who did not show regression. As 2 × 2 Fisher exact test subject numbers per cell were small, subjects who showed no change or worsened were collapsed into one group. The results revealed that the number of children without a history of regression did not statistically differ in terms of language change over time from the number of subjects who did not show regression.

**Table 9:** Time 1 to Time 2 Change in language scores for NSA group when comparing the patients with and without history of regression

	**Group**	**Better**	**NoDiff/worse**	**Fisher exact**
Receptive language				
	NoRegr	3	14	n.s.
	Regr	3	4	
Expressive language				
	NoRegr	7	10	n.s.
	Regr	3	4	

### Relationship between FMAER and language change scores for the STAR and NSA Groups separately

Table 10 shows the STAR group’s result of the stepwise multiple regression analysis performed in order to explore the relationship between changes in the FMAER and changes in language performance separately for receptive and expressive language across each subject’s two time points. The result was highly significant with just a single FMAER variable chosen for receptive language (FMAER at C3 left central region) and a single variable chosen for expressive language (FMAER at T7, left mid- temporal region). The first step in both analyses showed many of the 14 independent variables’ significant correlation with the dependent language measure (receptive 8/14, expressive 9/14 at F ≥ 4.0 to enter). However, at the second step after the removal of the effect of the first independent variable from the remaining 13 independent variables, none of the variables reached the F level ≥ 4.0; therefore none was chosen. This indicates suppression of 13 variables by the first variable chosen which in turn demonstrates that, as expected, that all 14 independent variables contain similar information. Table 11 shows the analogous result for the NSA group. Again the result was highly significant with a single variable (FMAER at P4, right parietal lobe) chosen for both the receptive and the expressive language scores. Again there was only one step, since the first variable chosen suppressed the remaining 13 variables due to the high shared information, i. e. high correlation among the variables.

**Table 10:** Multiple regression, STAR group: 14 FMAER differential FFT scores separately predict receptive and expressive language differential scores

**Variable entered**	**R**	**p**	**F to enter**
Receptive			
C3	0.6296	0.01	11.82
Expressive			
T7	0.6134	0.01	10.86

**Table 11:** Multiple regression, NSA group: 14 FMAER differential FFT scores separately predict receptive and expressive language differential scores

**Variable entered**	**R**	**p**	**F to enter**
Receptive			
P4	0.5909	0.01	11.81
Expressive			
P4	0.5288	0.01	8.54

Thus although similarly significant multiple FMAER-language regression scores were noted for both groups, the best FMAER-language correlation for the STAR group manifested itself in two left hemisphere electrodes (T7 receptive, C3 expressive) whereas for the NSA group the best language correlation manifested itself in a single right hemisphere electrode (P4 right parietal, for both, receptive and expressive).

### Change in behavioral criteria for autistic disorder STAR group

As shown in Table 12 the STAR group showed a highly significant reduction in the DSM-IV ASD scaled symptom summary scores when comparing the before and after treatment scores.

**Table 12:** Time 1 to Time 2 DSM-IV Score difference, STAR group

**Group**	**Average DSM-IV Score**	**d.f.**	**T**	**p**
t-test of mean of 14 scores (see text and Table 3)				
Before	2.0677	38	7.261	0.00001
After	0.9857			

### Complications of steroid treatment STAR group

As shown in Table 13 almost all STAR group subjects, as anticipated, demonstrated Cushingoid appearance and experienced weight gain associated with enhanced appetite. For all subjects weight and appearance returned to normal within several months of discontinuation of steroids. Excessive weight gain was typically managed successfully by parent-guided dietary restrictions, especially reduction of carbohydrate intake. Half of the population initially experienced behavioral worsening, typically irritability. This was usually successfully managed by a slight reduction of steroid medication. When severe (two subjects), risperdone therapy was used successfully without concurrent language deterioration. Only one subject required medical treatment for hypertension; the others responded to slight steroid reduction. Any observed GI bleeding and elevated serum calcium spontaneously normalized without need for intervention. The single urinary tract infection observed was easily treated with antibiotics. Two subjects with sleep disturbances (frequent wakening) were successfully treated with melatonin. Many subjects experienced cold weather associated upper respiratory tract infections without need for any special medical intervention. Two of the 20 subjects manifested slight language regression after conclusion of the steroid taper. Both responded well to 1–2 months of re-treatment using a pulse steroid dosing protocol (50% of prior weekly dose given one day a week for 1–2 months). None of the subjects developed cataracts. By approximately one year after cessation of steroid therapy those 17 subjects (Table 8) STAR subjects who had demonstrated clinical language improvement maintained and/or further improved their performance after cessation of treatment. The three subjects who had not responded well, manifested no significant change at the one-year follow-up point. However, this apparent stability was documented only by parent response.

**Table 13:** Treatment complications, STAR group


Cushingoid appearance: 18/20	Weight gain: 19/20
Significant hypertension: 2/20	High serum calcium: 1/20
GI bleeding: 1/20	Excess urine glucose: 1/20
Mild behavior disorder: 7/20	Severe behavior disorder: 3/20
Sleep disorder: 2/20	Infection: 1/20
Regression: 2/20	Cataracts 0/20

## Discussion

The overall goal of this study was to identify by retrospective data analysis, quantifiable evidence supportive of beneficial effects of adrenal corticosteroid (prednisolone) therapy on brain, language and behavioral function of children with a history of sudden autistic regression (R-ASD). Such evidence, while retrospective, nevertheless might serve to strengthen sufficiently the existing anecdotal reports for such salutary effects [15,24,25], in order to potentially justify a future prospective randomized trial of steroid treatment for R-ASD. To this end a large clinical patient database of children with a diagnosis of autism spectrum disorder (ASD) was searched for pertinent target subjects who met the criteria for regressive autism, were between the ages of 3 and 5 years at the time of their first FMAER study, had an absent or distorted initial FMAER study, received steroid treatment (STAR group), and had a second FMAER study performed after steroids were discontinued. Following identification of the target group subjects, a non-steroid-treated ASD comparison group was identified. These were comparable-age children also with an initial absent or distorted FMAER study and with a second FMAER study performed between 6 and 36 months later, yet not treated with steroids (NSA group). Twenty target group children (STAR) and 24 comparison group children (NSA) were identified (Table 4). The NSA group children were followed by their clinicians in the same overall time span as the STAR sample. Additionally available data aside from the two FMAER studies included for the STAR group receptive and expressive language scores based on a clinical assessment (CLSQ) specifically developed for pediatric ASD patients who receive pharmacological treatments, as well as DSM-IV ASD total number of symptom scores. For the NSA group receptive and expressive language scores derived from neurologists’ clinical notes were calculated for the two clinical visits temporally closest to the two FMAER tests utilized. All scores were available and had been entered into the clinical database prior to study design and analysis.

The investigation’s *first* and most important specific goal was to compare by means of objective spectral analysis, potential changes in the 4 Hz FMAER steady-state response between the first and second study point for each of the two subject groups separately. Results showed striking differences in change between the two populations (Table 5). For the STAR group, 9 of 14 electrodes showed markedly increased second-study 4 Hz response magnitudes with maximal effect in the left inferior-posterior temporal electrode, TP9 (p ≤ 0.00037). In contrast the NSA group failed to manifest any significant change in 4 Hz spectral power at any of the 14 electrodes Thus, the steroid treatment appears associated with a significant increase in the specific FMAER stimulation elicited 4 Hz response amplitude of the superior temporal gyri (STG) in both hemispheres of the study children with regressive autism. Additionally, the FMAER response distortion present at the first study point of the STAR group was absent at the second study point (Table 1) Thus, the STAR group demonstrated both higher amplitudes and a less distortion in the FMAER after steroid treatment.

Not all electrodes manifested a significant increase in the 4 Hz response to steroids in the STAR group (Table 5). The reason for this appears to lie in the fact that the FMAER may be modeled as a single dipole within each STG. These dipoles have both a specific physical location and a specific orientation. What is seen on the scalp is the projection of the dipole source from each STG upon the overlying scalp such that the scalp response magnitude reflects the dipole orientation and location which varies slightly among subjects. By including 14 distributed scalp locations seven per hemisphere, one may conclude that failure to demonstrate any significant changes in the NSA group did not arise from a failure to record over a ‘hot’ scalp region [16], but from the lack of a significant response change over time.

As Figure 1 illustrates the before and after spectral plots demonstrate that the subjects receiving steroids showed not only a significant increase in magnitude of the appropriate 4 Hz FMAER response but simultaneous that distortion products in the form of other than 4 Hz response components evident in the pre-medication FFT plots were significantly reduced. In their well-known speech quantification work, Wu and Pols [27] note that “…in many situations the speech quality is not satisfactory, often due to factors related to the transmission of the speech signal from speaker to listener. During transmission there is a variety of factors influencing the quality of speech, such as: a limitation of the frequency range or the dynamic range, noises, echoes, and other (analog or digital) distortion components.” On the basis of the current study’s results it appears reasonable to speculate that distortion may additionally occur within the central auditory pathway of the listener as visualized by the FMAER at the level of the STG.

It is hypothesized that language regression in autism may result from the development of dysfunction in the specific STG systems that are needed to accurately and cleanly detect rapidly changing spectral information within the acoustic stream. Children with R-ASD are not deaf or ‘hard-of-hearing’ *per se*; they often clearly identify even very soft novel sounds. The problem appears to reside in the auditory distortion that occurs within cortex devoted to language processing. This distortion appears to be at least partly reversible with steroid therapy.

The study’s *second* goal was to determine whether the significant FMAER improvements might be related to steroid-based suppression of presumed-to-be minor abnormalities in the children’s EEGs as observed by visual inspection. Note that neither group demonstrated frank epileptiform transients such as spikes or spike and wave patterns. The EEG change rankings did not differ between the two groups (Table 6). Additionally the EEG changes in the STAR group included both EEG improvement and EEG worsening. These findings emphasize that there appears to be little evidence for a physiologically meaningful steroid effect on EEG. This is an important finding that runs contrary to the assumption made in Landau-Kleffner syndrome, namely that both language and FMAER improvements with steroids result from suppression of frequent spike discharges within the STG [16]. What may be common to R-ASD and LKS children’s brains is dysfunction of the STG. However, the dysfunction need not necessarily be accompanied by spike discharges. It may, none-the-less, be ameliorated by steroids.

The *third* goal was to compare changes in clinical-rating-based language scores between the first and the second study. As shown in Table 7 the STAR group showed highly significant improvement in the language ratings between time study 1 and 2; the improvement was comparable for both the receptive and expressive language ratings. Moreover significantly more STAR group subjects (17/20) than NSA group subjects showed improvement (6/24 ‘better’ receptive, and 10/24 ‘better’ expressive). These data suggest that steroid treatment may be associated with improvement in language and that more subjects who receive steroid treatment may show such improvement than subjects in the non-treated group.

In order to rule out the possibility that regressive autism spontaneously improves with time estimated language change of the seven NSA group children with histories of regression was compared to that of the 17 NSA group children without such a history of regression (Table 4). Although the small population size precludes a definitive answer, there was no significant difference between the two NSA subpopulations for receptive or for expressive language ratings (Table 9). Thus, spontaneous NSA group improvement of language in the regressive autism subpopulation was not observed.

The *fourth* goal was to explore the possible relationships between changes in 4 Hz spectral responses at all electrodes and the language ratings change scores (Table 7) for receptive and expressive language separately. The STAR group (Table 10) showed a strong correlation between receptive language and the FMAER at the left central region (C3) and between expressive language and the FMAER in the left mid temporal region (T7). It appears clinically meaningful that STAR group language improvement was more evident in the left hemisphere as the left hemisphere is typically the dominant hemisphere for language. Given that the scalp FMAER data are highly inter-correlated and reflect activity at the level of the STG, the difference in the scalp location of maximal effect for correlations with receptive and expressive language is curious. It is possible that slightly different regions of the STG are responsible for expressive and receptive language functions resulting in subtle differences in the dipole source orientations or locations and correspondingly different in patterns of scalp projection. This is an area for future exploration. For the NSA group (Table 11) a strong correlation was found between the right parietal (P4) region and both the receptive and the expressive language scores. It is curious that the typically non-language dominant right hemisphere STG maximally correlated with NSA group language function. This may reflect relatively greater dysfunction within the left hemisphere in this group. These findings are limited by the clinical rating measures of language employed in this study. Formal language testing with standardized assessments will be required to substantiate these preliminary findings.

The *fifth* goal was to determine the level of behavioral improvement within the steroid-treated group. The scaled DSM-IV symptom summary scores demonstrated a highly significant improvement after steroid treatment (p ≤ 0.00001) (Table 12) Thus steroid treatment may be associated with behavioral improvement.

Finally the study’s *sixth* goal was to weigh the complications of steroid administration against their potential benefits (Table 13). Overall, almost all steroid-treated subjects gained weight. Working with families to counteract increased appetite and associated weight gain was a dominant clinical task in patient management. Behavior and sleep disorders were readily managed pharmacologically. The two subjects, who experienced behavioral regression after steroid taper, were successfully treated with a short period of pulse dosing. There were few serious complications although the process clearly increased the task of managing the child’s well-being and health for the parent and should only be undertaken with comprehensive, detailed understanding of the ramifications involved in such a significant medication trial. The most frequent management tasks for the parents were: (1) the constant need to monitor food intake in order to avoid weight gain which in turn might facilitate development of secondary hypertension; and (2) the difficulty in managing the child’s behavior.

It is unclear at this point how steroids might function to improve language and behavior in regressive autism. Aside from a relatively sudden clinical onset the current study fails to establish similarities between R-ASD and the Landau-Kleffner syndrome and other epileptic syndromes. Some have suggested an immune process, possibly initiated by outside factors that challenge the immune system. Only two of the 20 families in the STAR-group suggested regression onset in close relationship to inoculations (MMR, influenza vaccination). Extensive studies reported in the literature have failed to identify a clear association between autism (including R-ASD) and protective immunizations [38]. Eighteen of the 20 current study’s R-ASD STAR group subjects provided no clinical information as to possible trigger events. Although a question of importance, the current data do not shed light on regression etiology.

As reviewed by Kurian and Korff [18] corticosteroids, although not necessarily a first-line treatment, have been used with some success in a number of epileptic syndromes aside from LKS. These include infantile spasms, Doose syndrome, Dravet syndrome, Rasmussen’s encephalitis, absence epilepsy, and acute ‘symptomatic’ seizures. There is, unfortunately, no agreement as to the mechanism(s) of corticosteroid actions as an anticonvulsant. However, a study by Di Chiara et al. [31] found in a rat model that glucocorticoids may simultaneously influence different pathways that differentially facilitate inhibitory GABA release and inhibit excitatory glutamate release, thus summing to an overall effect of neuronal suppression. Relevancy to epilepsy, LKS and R-ASD is merely speculative at this point. As reviewed by Chatham and Kimberly [39], corticosteroids manifest a well-documented multiplicity of cellular effects that may be relevant to treatment of immune disorders such as lupus. Again, the relevancy to R-ASD remains to be established.

Hopefully a better understanding of corticosteroid action mechanisms may lead to the development a more precisely targeted drug that has fewer secondary complications and is useful in epilepsy treatment as well as the amelioration of R-ASD symptomatology.

The current study benefits from the availability of the simple and objective FMAER test that is relevant not only for detection of response amplitude changes but also of response distortion. Nevertheless the current study has very significant limitations that preclude conclusions as to the effectiveness of steroid treatment for regressive autism. The study, however, appears to provide sufficient indication in support of the need for a prospective, randomized, double blind, placebo controlled, cross-over design trial in order to assess more rigorously the potential role of steroids in R-ASD.

Study limitations as indicated above include but are not limited to the following:

(1) The study was retrospective.

(2) The STAR and NSA groups were similar yet did not meet all of the same selection criteria. The STAR group was composed exclusively of children with regressive autism while the NSA group was composed mostly of children with autism without history of regression.

(3) Aside from the FMAER different language measures were applied to STAR and NSA group. Neither of the language measures is standardized or published.

(4) Quantification of the DSM-IV symptom list as a measure of behavior is also unpublished and not in standard usage; moreover this measure was only available for the STAR group.

(5) Analysis by ANOVA with interaction and possibly with covariates would be preferred over paired T-test if the groups were more comparable initially.

(6) Aside from the FMAER the language and behavior data were obtained by clinicians in collaboration with the parents rather than by independent observers.

(7) The one year follow-up is inadequate in terms of length of time from treatment and in terms of the rigor of the language and behavior data gathered.

## Conclusion

Children with R-ASD, and without concurrent evidence for an epileptic encephalopathy such as the Landau-Kleffner syndrome, who receive steroid therapy, show improvement in a language specific electrophysiological brain function indicator, as measured with the FMAER. Additionally, they appear to show improvement in language and behavior performance. At the level utilized in this study, steroid therapy did not appear to result in recognized, lasting morbidities.

The current study augments prior demonstrations of the utility of the FMAER as a quantified and objective tool for the study of childhood language disorders [16]; the FMAER appears sensitive not just to the presence or absence of a response and to response magnitude but also to response distortion, the latter being prominent in R-ASD. Although the current study goes beyond single case reports in suggesting a positive steroid response in regressive autism, it mainly serves to support the need for a more formal, prospective study as noted above.

The current study does *not* suggest that steroids ‘cure’ regressive autism nor does it claim proof of the value of pharmacological treatment of regressive autism.

The following questions are left unanswered and require future investigations:

(1) Will a formal prospective randomized, double blind, placebo controlled, crossover study validate current results?

(2) Will results differ if steroids were delivered sooner after regression rather than several months later as was the case for the current study?

(3) How will steroid therapy compare to anticonvulsant therapy or other medication trials in ameliorating R-ASD?

(4) Will the beneficial effects of steroids endure beyond a year?

(5) What will be the implications of a positive steroid response to the hypothesis of an inflammatory nature of regression in ASD?

(6) Will early MEG and/or dense EEG array studies of R-ASD patients demonstrate epileptiform discharges not observed by EEG?

(7) Will steroids play a role in the treatment of non-regressive autism?

(8) Will distortion of the FMAER be demonstrable in forms of central auditory processing syndromes other than regressive autism?

## Abbreviations

ASD: Autism spectrum disorder; BCH: Boston Children’s Hospital; CLSQ: Clinical language status questionnaire; DNL: Developmental neurophysiology laboratory; DSM: Diagnostic and Statistical Manual; EEG: Electroencephalogram / electroencephalographic; FMAER: 4 Hz frequency modulated auditory evoked response; FFT: Fast Fourier transform; GDD: Global developmental delay; Hz: Hertz or cycles per second; LKS: Landau-Kleffner syndrome; MEG: Magnetoencephalogram/magnetoencephalography; MMR: Measles, mumps, rubella vaccine; NSA: Non-steroid treated group; R-ASD: Regressive ASD; STAR: Steroid treated autism with regression group; STG: Superior temporal gyrus/gyri; μV: Microvolt.

## Competing interests

The authors declare that they have no competing interests.

## Authors’ contributions

All authors contributed to the study’s concept and design including the range of topics to be analyzed and reported. FHD and HA conceived of the study design with significant assistance and advice from YZ, DC, and AR. FHD supervised all aspects of the study. FHD and AS selected the subjects to be included in the study. FHD was responsible for the acquisition, preparation and analysis of all neurophysiologic data including the clinical EEG readings. FHD finalized CLSQ development although all authors and additional clinicians were involved in its conception, reliability assessment, and use. FHD and AS abstracted the language and behavioral data from the records. FHD and GBM performed the statistical analyses. FHD developed the software needed for and performed the noise analyses. FHD had full access to all data in the study and takes responsibility for all aspects of the study including integrity of data accuracy and data analysis. FHD, AS, and HA collaborated in writing and editing the paper. FHD and HA responded to the reviewers and wrote and edited the revised versions. All authors approved the final manuscript.

## Authors’ information

FHD is a physician, child neurologist, electroencephalographer, neurophysiologist and epileptologist with undergraduate degrees in electrical engineering and mathematics. His current research interests are in neuro-developmental disorders (including autism) and epilepsy, including the development and utilization of specialized techniques to support related investigations. AS is a cognitive neuroscientist with specialized interests in EEG and evoked potentials and their identification of neuro-developmental disorders, particularly disorders of language. GBM is a neuropsychologist and statistician with specific interests in pediatric neurodevelopment. YZE is a physician, child neurologist, epileptologist, electroencephalographer, and clinical and research neurophysiologist. DC is a physician and child neurologist with specialized training and interests in the intellectual disabilities (including autism) and cerebral palsy including interests in newer methods for diagnosis and treatment. AR is a physician, child neurologist, epileptologist, electroencephalographer, and clinical and research neurophysiologist. His research interests include transcranial magnetic stimulation and its application to epilepsy and neurodevelopmental disorders. HA is a research, developmental and clinical psychologist with interest in infant and child neurodevelopment, including generating predictors of later outcome from neurophysiologic data.

## Pre-publication history

The pre-publication history for this paper can be accessed here:

http://www.biomedcentral.com/1471-2377/14/70/prepub
